# Ambulatory Care Preferences of Older People Age 80 and Over: A Systematic Review of Qualitative Studies

**DOI:** 10.1093/geroni/igaa057.640

**Published:** 2020-12-16

**Authors:** Angélique Herrler, Helena Kukla, Vera Vennedey, Stephanie Stock

**Affiliations:** 1 University of Cologne, Cologne, Germany; 2 Universital Hospital of Cologne, Cologne, Germany

## Abstract

According to the United Nations, the number of people aged 80 and over is expected to treble by 2050 globally. But research on the preferences for care of this age group grows slowly. To achieve high-quality patient-centered care, we need to understand the oldest people’s specific living circumstances, care preferences and goals. The aim of the study was to synthesize findings about ambulatory care preferences, experiences and expectations of people aged 80 and over. We systematically searched Medline, CINAHL, PsycInfo, Web of Science Core Collection and Google Scholar for qualitative studies published until October 2019 and additionally conducted forward and backward citation search for included studies. Two independent reviewers assessed studies for eligibility criteria and quality. We performed a thematic synthesis of study findings as developed by Thomas and Harden using MAXQDA-20 content analysis software. Twenty-three studies were included. They were mainly conducted in Europe, used face-to-face interviews, reported on ambulatory home care and used qualitative content or thematic analysis. The meta-synthesis revealed two fundamental themes from the perspective of older people: feeling safe and feeling valued in their relationships with caregivers and in their care environment. This was shown, for instance, in preferences for coordinated care, high continuity of caregivers, personal attention and interactions based on trust and respect. In practice, the older persons’ preferences should be integrated into care planning and policies to ensure patient-centered care.

